# Pelvis Morphology, Trunk Posture and Standing Imbalance and Their Relations to the Cobb Angle in Moderate and Severe Untreated AIS

**DOI:** 10.1371/journal.pone.0036755

**Published:** 2012-07-05

**Authors:** Georges Dalleau, Pierre Leroyer, Marlène Beaulieu, Chantal Verkindt, Charles-Hilaire Rivard, Paul Allard

**Affiliations:** 1 Faculté des Sciences de l’Homme et de l’Environnement, CURAPS-DIMPS, Université de la Réunion, Le Tampon, France; 2 Department of Kinesiology, University of Montreal, Montreal, Parti Québécois, Canada; 3 Department of Orthopedic Surgery, Sainte-Justine Hospital, Montreal, Parti Québécois, Canada; 4 Human Movement Laboratory, Research Centre, Saint-Justine Hospital, Côte Sainte-Catherine, Montreal, Quebec City, Canada; Universidad Europea de Madrid, Spain

## Abstract

Adolescent idiopathic scoliosis (AIS) is the most common form of scoliosis and usually affects young girls. Studies mostly describe the differences between scoliotic and non-scoliotic girls and focus primarily on a single set of parameters derived from spinal and pelvis morphology, posture or standing imbalance. No study addressed all these three biomechanical aspects simultaneously in pre-braced AIS girls of different scoliosis severity but with similar curve type and their interaction with scoliosis progression. The first objective of this study was to test if there are differences in these parameters between pre-braced AIS girls with a right thoracic scoliosis of moderate (less than 27°) and severe (more than 27°) deformity. The second objective was to identify which of these parameters are related to the Cobb angle progression either individually or in combination of thereof. Forty-five scoliotic girls, randomly selected by an orthopedic surgeon from the hospital scoliosis clinic, participated in this study. Parameters related to pelvis morphology, pelvis orientation, trunk posture and quiet standing balance were measured. Generally moderate pre-brace idiopathic scoliosis patients displayed lower values than the severe group characterized by a Cobb angle greater than 27°. Only pelvis morphology and trunk posture were statistically different between the groups while pelvis orientation and standing imbalance were similar in both groups. Statistically significant Pearson coefficients of correlation between individual parameters and Cobb angle ranged between 0.32 and 0.53. Collectively trunk posture, pelvis morphology and standing balance parameters are correlated with Cobb angle at 0.82. The results suggest that spinal deformity progression is not only a question of trunk morphology distortion by itself but is also related to pelvis asymmetrical bone growth and standing neuromuscular imbalance.

## Introduction

Scoliosis is a three-dimensional (3D) deformation of the spine and rib cage resulting in a prominent trunk distortion. Its more common form is adolescent idiopathic scoliosis (AIS) usually affects young girls [1]. Most studies describe the differences between scoliotic and non-scoliotic girls and focus primarily on a single set of parameters derived from spinal and pelvis morphology [2], posture [3], [4] or standing imbalance [5], [6]. Few addressed a combination of different types of parameters, for instance, curve type and postural sway [7] or body posture and standing stability [8] in AIS. Fewer reported differences in untreated adolescent idiopathic scoliosis of different severities for standing balance [9], [10] and pelvis morphology asymmetries [11]. No one as yet combined all three aspects in a single study and their relation to spinal deformity progression in untreated AIS of different severity.

Predominance of patients with a large Cobb angle, which is the most widely used scoliosis descriptor, and of its progression, could explain in part the differences observed with non-scoliotic girls. In posture and standing balance studies involving a single scoliotic group, the Cobb angle varies widely: 5° to 52° in Nault et al. [8], 6° to 86° in Nicolopoulos et al. [12] and 10° to 45° in Pasha et al. [13]. When several scoliotic scoliotic groups were compared the maximum Cobb angle within each group was close to 40° [2] or higher [7] and included patients scheduled for surgery. Often studies include more than a single curve type [4] or combine different form of scoliosis such as adolescent idiopathic scoliosis, infantile idiopathic scoliosis and scoliosis associated with another condition and back problems [14]. This makes it difficult to appreciate if differences between girls typically developing scoliosis and able-bodied girls are due to the severity of spinal deformity or its progression.

Correlations between the Cobb angle and radiographic, morphologic, postural and standing balance parameters met mitigated successes [15]. Nonetheless, Ramirez et al. [16] reported a coefficient of correlation of 0.7 for scoliometer readings while Goldberg et al. [14] found value of 0.8 between the Quantec spinal angle obtained from topographic scans. Both these studies underline the importance of trunk morphology distortion resulting from the spinal deformity but do not address the implications of standing imbalance and bone growth in relation to the Cobb angle progression.

No one has assess within the same study pelvis morphology, asymmetrical posture and standing imbalance in pre-braced AIS girls of different scoliosis severity but with similar curve type and the interaction of these factors with scoliosis progression. The underlying hypothesis of this study is that the Cobb angle is related not only to trunk morphology but also to a complex relationship between asymmetrical bone growth [17], [18] and neuromuscular control [19] as well. The first objective is to test if there are differences in these parameters between pre-braced AIS girls with a right thoracic scoliosis of moderate (less than 27°) and severe (more than 27°) deformity. The second objective is to identify which of these parameters are related to the Cobb angle progression either individually or in combination of thereof.

## Method

**Figure 1:**
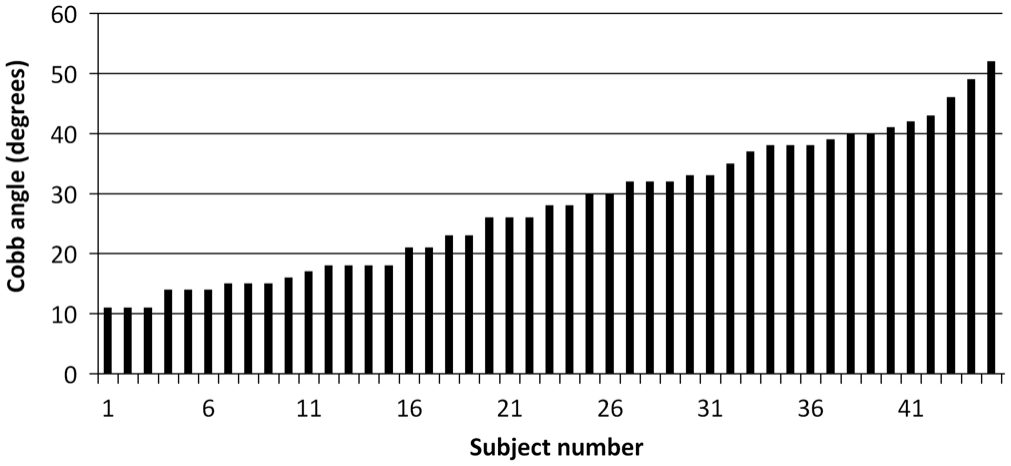
Cobb angle values of all subjects listed in increasing order.

In all, 45 scoliotic girls were randomly selected by an orthopedic surgeon from the hospital scoliosis clinic based on the definition given by Bunnell [20]. Their average Cobb angle was 28° ±11° and ranged between 11°–52°. No subject was under active treatment and all curves were to the right. Their average age was 12.6±1.6 years while their height and mass were 153.6±9.7 cm and 43.4±9.0 kg, respectively. They were divided into a group of moderate and severe untreated scoliosis. The division point was a Cobb angle of 27° corresponding to the median of the group. This is justified considering that curves greater than 25° are often considered as severe [20], [21], [22], [23], [24]. Any subject wearing a foot orthosis, having a limb length discrepancy of more than 1 cm, displaying any other signs of postural orthopedic or neurological disorders were excluded from the study. Fig. 1 illustrates the Cobb angle in increasing order for all scoliotic subjects. It represents a regular progression from 11° to 52° indicating a continuous pattern without any predominant Cobb angle value.

The mean demographic characteristics of the pre-braced moderate and severe scoliotic groups are given in Table 1. There was no statistical difference between the groups in terms of age (p = 0.38), height (p = 0.10) or mass (p = 0.29). Prior to the experimentation, all procedures were explained to each subject and his parents who signed a written consent. This study and the consent procedure were approved by the Sainte-Justine Hospital Ethics Committee.

**Table 1:** Mean values and standard deviations for age, height, mass and Cobb angle and range are given for the moderate and severe scoliosis groups.

Group	n	Age (years)	Height (m)	Mass (kg)	Cobb angle (deg)	Cobb angle range (deg)
Moderate scoliosis	22	12.4±1.8	1.51±0.09	41.9±8.0	17.8±4.7	11–26
Severe scoliosis	23	12.8±1.4	1.55±0.10	44.8±9.8	37.2±6.5	28–52

Two types of measurements were taken. For pelvis morphology and for trunk posture ten bony landmarks were identified on the subject standing barefoot with the heels spaced by 23 cm and the feet pointing externally by 15° [25]. Pelvis morphology is defined by the right and left anterior (ASIS) and posterior (PSIS) superior iliac crests as well as the first sacral (S1) vertebra [13]. The trunk spatial orientation is given by S1 and the seventh cervical (C7) spinous processes and the right and left acromions and inferior angles of the scapula [8].

While subjects maintained a quiet stance, the operator lightly touched the skin lying over the anatomical landmarks with the tip of the pen of a Flock of Bird system (Ascencion Technologies, Burlington, VT, USA) to register their 3D coordinates. Such as system was used by Leblanc et al. [26], Nault et al. [8] and Stylianides et al [11] to study pelvis morphology body and trunk posture in AIS. Bellefleur et al. [27] estimated the linear and angular resolution of the electromagnetic system to be 0.76 mm and 0.1° respectively. These values are within those reported for video-based systems where 10 mm in diameter surface markers are used to assess pelvis and trunk motions in standing [13].

The three-dimensional coordinates of these bony landmarks were calculated with respect to S1 of each subject with positive axes to the right, anterior and upward. The mean right and left pelvis width correspond to the average distance of the ASIS and PSIS from S1 for each side. Similarly mean pelvis depth and height are the average of the ASIS and PSIS measured from S1. Increases in these dimensions are indicative of pelvis growth. Pelvis sagittal tilt, frontal tilt and transverse orientation are determined as describe by Pasha et al. [13] using the pelvis anatomical landmarks. The trunk sagittal and frontal inclinations are calculated by the angle sustained by the line joining S1 to C7 and the vertical. Because of the trunk distortion the lower trunk posture defined by the line joining S1 to scapula midpoint (S1-Sca) and that of the upper trunk given by that of the scapula midpoint to C7 (Sca-C7) were also calculated with respect to the vertical. The trunk transverse orientations are determined by the angle sustained by the line joining the acromions and the transverse axis and that of the line joining the inferior angle of the scapula and the transverse axis.

The second set of measurements characterized the quality of quiet standing. Each subject stood on an AMTI force plate (Model OR6-5, Newton, MA) with the feet positioned as described above. With the arms parallel to the trunk the subject focused on a target placed at 1.2 m ahead and located at eye level [5], [28]. Three trials of 64 s were performed at a sampling frequency of 64 Hz [5], [8]. The anterioposterior (AP) and mediolateral (ML) center of pressure (COP) excursions were calculated from forces and moments. Afterwards the COP range which is the difference between the maximal and minimal COP values and the COP speed (sum of COP displacements/64 s) in both AP and ML directions were calculated for each trial of a subject and then respectively averaged. The amplitude of the COP range is suggestive of standing imbalance [29] while that of the COP speed corresponds to the neuromuscular system demand [30] (Maki et al., 1994) to avoid loss of balance. The free moment (Tz) is representative of an asymmetric control of the trunk around the vertical axis during standing balance [9], [31] and is expressed in Nm. It was normalized with respect to the subject body mass (Nm/kg). Then the mean, range and RMS values of the normalized free moment were also calculated for the duration of each trial of a subject and respectively averaged.

Multivariate analyses (Manova) were used for statistical comparisons for the pelvis morphology and orientation, trunk posture and balance parameters. Whenever the Manovas reached a significant level, planned comparisons were used to examine the specific effect. A Bonferroni correction procedure was applied to control Type 1 error by adjusting the p values in the analysis of the aforementioned parameters [32]. For all statistical analyses a p value equal or less than 0.05 was considered as significant. Pearson coefficients of correlation were performed to identify any relationships between Cobb angles and the 24 pelvis morphology, trunk posture and balance parameters. Furthermore a stepwise multiple regression analysis was performed to determine if the coefficient of correlation could be improved with any combination of the above mentioned parameters using a p value of 0.05.

## Results

The mean and standard deviations of the pelvis morphology, trunk posture and balance parameters for the moderate and severe scoliosis groups are given in Table 2. Generally these parameters were more pronounced in the severe scoliotic group. The Manovas revealed a statistical difference between the moderate and severe scoliotic groups for the pelvis morphology (p = 0.01) and trunk posture (p<0.001) only. Further analyses revealed statistically significant differences that the mean right and left pelvis widths were 9 mm (11%) longer in the severe group. Though the S1-C7 balance or alignment was nearly vertical, the severe scoliotic group had a significant 3° S1-SCA greater inclination to the right and a similar SCA-C7 left compensation. The trunk sagittal inclinations were statistically more pronounced by also 3° with a 5° greater rotation of the acromions in the severe scoliotic group. There was no significant difference in the pelvis orientation (p = 0.37) and both groups behaved similarly in standing balance (p = 0.67).

**Table 2:** Mean and standard deviation of the pelvic morphology, truck posture and standing balance parameters.

	Moderate	Severe	p value
Pelvis morphology			
Width right pelvis (mm)	67.4±7.2	76.0±6.6	<0.001*
Width left pelvis (mm)	−75.7±11.7	−75.4±12.5	0.93
Height right pelvis (mm)	26.4±15.3	27.9±14.6	0.74
Height left pelvis (mm)	20.6±17.3	21.7±14.3	0.81
Depth right pelvis (mm)	82.5±12.4	81.6±11.3	0.82
Depth left pelvis (mm)	78.7±9.5	87.2±9.3	<0.01*
Pelvis orientation			
Pelvis rotation (degree)	1.9±7.1	−2.1±7.8	0.08
Pelvis frontal tilt (degree)	2.2±3.2	2.0±2.8	0.83
Pelvis sagittal tilt (degree)	6.3±9.4	6.0±7.5	0.89
Trunk posture			
S1-C7 frontal (degree)	−1.1±2.0	−0.4±3.4	0.41
S1-Sca frontal (degree)	−0.3±3.7	2.7±5.2	0.03*
Sca-C7 frontal ((degree)	−2.2±2.8	−5.1±4.2	<0.01*
S1-C7 sagittal (degree)	3.5±2.3	5.4±2.6	0.02*
S1-Sca sagittal (degree)	−1.2±3.3	1.8±4.8	0.02*
Sca-C7 sagittal ((degree)	10.3±5.0	11.1±4.3	0.59
Scapula rotation (degree)	3.4±5.4	4.2±5.3	0.59
Acromion rotation (degree)	0.6±6.5	−5.0±7.5	0.01*
Standing balance			
COP range ML (mm)	16.6±7.5	21.0±9.2	0.09
COP speed ML (mm/s)	7.6±3.0	8.9±4.0	0.21
COP range AP (mm)	28.6±7.5	32.1±13.5	0.29
COP speed AP (mm/s)	9.8±2.8	10.3±2.1	0.54
Tz mean (Nm/kg)	0.283±0.079	0.256±0.134	0.40
Tz range (Nm/kg)	0.359±0.239	0.378±0.234	0.79
Tz RMS (Nm/kg)	0.036±0.017	0.053±0.064	0.21

The star (*) denotes a significant difference.

The Pearson coefficients of correlation were performed between the Cobb angle and each pelvis morphology and orientation, and trunk posture parameters. The right pelvis width displayed the highest significant coefficient at 0.54 (p<0.001) while the second highest was with the left pelvis depth at 0.425 (p = 0.008). Five of the trunk postural parameters had statistically significant correlations. In the frontal plane these were the angle between the vertical and S1-Sca (r = 0.320, p = −0.068), Sca-C7 (r = 0,386, p = 0.007) and for the sagittal plane the significant correlations were for the S1-C7(r = 0.356, p = 0.027) and S1-Sca (r = 0.356, p = 0.004). Only the acromial rotation angle was significant (r = 0.376, p<0.001). Standing balance parameters were not significantly correlated with the Cobb angle with the highest value at 0.26.

A stepwise multiple regression analysis increased the maximum r value from 0.59 to 0.83 (p = 0.08). Since it was not statistically significant each group of parameters (pelvis morphology and orientation, trunk posture and balance) was in turn tested separately. The resulting r values are given in Table 3. Only the trunk posture and pelvic morphology data sets had a significant r value. The highest correlation was obtained by using simultaneously the 8 trunk posture parameters resulted in an r value of 0.71. Analysis was also performed by keeping any two sets of parameters. The results are given in Table 4. The best combinations were obtained with trunk posture and any of the three other data sets. These were all above 0.71 but the highest correlation was obtained with pelvis morphology (0.77). Combining three data sets further improved the correlation as shown in Table 5. The highest correlation with the Cobb angle (r = 0.82) was with trunk posture, pelvic morphology and balance parameters.

**Table 3:** Coefficients of correlation for a stepwise multiple regression analysis using pelvis morphology and orientation, trunk posture and standing balance.

Parameter	R	*p*
Trunk posture	0.710	<0.001*
Pelvis morphology	0.569	0.01*
Balance	0.369	0.57
Pelvis orientation	0.346	0.15

The star (*) denotes statistically significant r values.

**Table 4:** Coefficients of correlation for a stepwise multiple regression analysis using any two combinations of pelvis morphology and orientation, trunk posture and standing balance.

Parameter 1	Parameter 2	R	*p*
Trunk posture	Pelvis morphology	0.775	<0.01*
Trunk posture	Balance	0.754	0.01*
Trunk posture	Pelvis orientation	0.718	<0.01*
Pelvis morphology	Balance	0.613	0.19749
Pelvis morphology	Pelvis orientation	0.601	0.04*
Pelvis orientation	Balance	0.484	0.43073

The star (*) denotes statistically significant r values.

**Table 5:** Coefficients of correlation for a stepwise multiple regression analysis using any three combinations of pelvis morphology and orientation, trunk posture and standing balance.

Parameter 1	Parameter 2	Parameter 3	R	p
Trunk posture	Pelvis morphology	Balance	0.82	0.03*
Trunk posture	Pelvis morphology	Pelvis orientation	0.79	<0.01*
Trunk posture	Pelvis orientation	Balance	0.76	<0.05*
Pelvis orientation	Pelvis morphology	Balance	0.65	0.28

The star (*) denotes statistically significant r values.

## Discussion

This study evaluated if pelvis morphology and orientation, trunk posture and standing balance was affected by the degree of spinal deformity in untreated scoliotic girls. Noticeable differences were reported between non-scoliotic and scoliotic AIS patients by numerous authors [4], [7], [8], [28], [31] but often these studies included scoliotic girls with severe scoliosis or girls treated or scheduled for treatment. To our knowledge only Haumont et al. [10] and Stylianides et al. [11] included a group of untreated scoliotic patients with mild or moderate severity respectively. This study differentiates itself from the later by including trunk posture to balance and pelvis morphology. Generally the girls with moderate scoliosis display smaller deviations in pelvis and trunk morphology and standing balance and these values were closer to those reported for able-bodied girls [4], .

Skeletal disproportion is associated with scoliosis [26], [33], [34] supporting an altered skeletal growth [11], [12], [17]. Our findings support these studies on asymmetrical pelvis growth characterized by a wider right pelvis and a deeper left pelvis in scoliotic girls with a severe spinal deformity. There was no difference in the pelvis orientation between the scoliotic groups. This can be explained in part by the large standard deviations resulting from pelvis deformation [11].

Our study is the first to describe trunk and pelvis orientations in moderate and severe pre-braced scoliotic girls. The results for the severe scoliotic group are similar to those of previous studies. Nault et al. [8] and Zabjek et al. [4] reported that AIS girls displayed greater deviations from normal in the postural measures illustrating an asymmetric posture particularly in the horizontal plane. But more importantly, our results underline that these observations are mostly related to the severe form of untreated scoliosis. Patients with curves of less than 27° should not be included with patients with a large Cobb angle when compared to an able-bodied group.

Differences in standing imbalance between AIS girls of different scoliosis severity were previously reported. Beaulieu et al. [9] have shown that girls under observation have a better postural control than those for which a Boston body brace was prescribed. This was later confirmed by Haumont et al. [10]. These studies imply that standing instability is either not present or has little effect in girls with mild scoliosis. Our results also indicate a greater postural control in girls with moderate scoliosis but not statistically different from those with a severe scoliosis. The lack of significant difference in the balance parameters between the moderate and severe pre-braced scoliotic group and their low coefficients of correlation with Cobb angle imply a later manifestation or a slow progressive deterioration. This also support our hypothesis that standing imbalance occurs in AIS but after the on-set of the scoliosis.

Simple linear regressions relating Cobb with body morphology met varying successes. Samuelson and Norén [35] and Amendt et al. [36] found coefficients ranging from 0.46 to 0.65 between Cobb angle and scoliometer readings. Using both clinical and surface topographic features Ramirez et al. [16] found similar coefficients of correlation for cosmetic score which included shoulder angles, scapula angle and trunk twist. Our statistically significant coefficients of correlation are within the range reported by Mubarak et al. [37] but included pelvis growth supporting that a horizontal asymmetrical pelvis growth [18] could result in scoliosis [11]. Using a topographic system Goldberg et al. [14] reported coefficient of correlation of 0.80. These results based on patients having a predominance of severe scoliosis underline the importance of trunk distortion resulting from the spinal deformity.

Stepwise multiple regression analysis provided higher coefficients of correlation and underlined the combined effect of pelvis growth, trunk posture and standing balance. In other words scoliosis is not only a question of asymmetrical trunk morphology by itself but is also related to asymmetrical bone growth and the neuromuscular control of standing balance. These parameters were previously identified but individually. Lonstein and Carlson [22] and Dickson and Sevitt [38] identified age and growth as factors of spinal deformity progression while Burwell et al. [17] Gum et al. [18], Stylianides et al. [11] associated asymmetrical pelvis growth to scoliosis. Leblanc et al. [26] and Zabjek et al. [4] reported asymmetrical pelvis and trunk postures in AIS while Simoneau et al. [6], [29] found scoliosis associated to a sensory deprivation and perturb balance control. Though these differences were noted no one had beforehand established their relationship with scoliosis progression or their interactions. It was by combining three sets of parameters that the highest coefficients of correlations were obtained considering the lack of statistical difference between our scoliotic groups and their weaker contribution to the overall correlation with the Cobb angle.

The results of the present study could be restrictive since no definite conclusion can be drawn about the causal relationship between the Cobb angle and abnormal trunk and pelvic morphology and standing imbalance in AIS. A study that includes a longer follow-up period is required to infirm the influence of changes in these parameters on the Cobb angle. However these findings provide a first attempt in combining different morphological and standing balance control to assess spinal deformity progression in AIS. These results justify in part a prospective radiographic study on pelvic growth of girls typically developing scoliosis in addition to monitoring their spinal deformity.

### Conclusion

This is the first study that compares a moderate and severe group of untreated idiopathic scoliosis patients using pelvis morphology and orientation, trunk posture and standing balance parameters. Generally moderate pre-brace idiopathic scoliosis patients displayed lower values than the severe group characterized by a Cobb angle greater than 27°. Only pelvis morphology and trunk posture were statistically different between the groups while pelvis orientation and standing imbalance were similar in both groups. Statistically significant Pearson coefficients of correlation ranged between 0.32 and 0.53 and were within the reported values. Using stepwise multiple regression analysis the correlations increased to 0.82. This suggests that spinal deformity progression is not only a question of trunk morphology distortion by itself but is also related to pelvis asymmetrical bone growth and standing neuromuscular imbalance.

## References

[1] Weinstein S, Weinstein SL (1994). Adolescent idiopathic scoliosis: Prevalence and natural history..

[2] Mac-Thiong JM, Labelle H, Charlebois M, Huot MP, de Guise JA (2003). Sagittal plane analysis of the spine and pelvis in adolescent idiopathic scoliosis according to the coronal curve type.. Spine (Phila Pa 1976).

[3] Masso PD, Gorton GE 3rd (2000). Quantifying changes in standing body segment alignment following spinal instrumentation and fusion in idiopathic scoliosis using an optoelectronic measurement system.. Spine (Phila Pa 1976).

[4] Zabjek KF, Leroux MA, Coillard C, Rivard CH, Prince F (2005). Evaluation of segmental postural characteristics during quiet standing in control and Idiopathic Scoliosis patients.. Clin Biomech (Bristol, Avon).

[5] Allard P, Chavet P, Barbier F, Gatto L, Labelle H (2004). Effect of body morphology on standing balance in adolescent idiopathic scoliosis.. Am J Phys Med Rehabil.

[6] Simoneau M, Richer N, Mercier P, Allard P, Teasdale N (2006). Sensory deprivation and balance control in idiopathic scoliosis adolescent.. Exp Brain Res.

[7] Gauchard GC, Lascombes P, Kuhnast M, Perrin PP (2001). Influence of different types of progressive idiopathic scoliosis on static and dynamic postural control.. Spine (Phila Pa 1976).

[8] Nault ML, Allard P, Hinse S, Le Blanc R, Caron O (2002). Relations between standing stability and body posture parameters in adolescent idiopathic scoliosis.. Spine (Phila Pa 1976).

[9] Beaulieu M, Toulotte C, Gatto L, Rivard CH, Teasdale N (2009). Postural imbalance in non-treated adolescent idiopathic scoliosis at different periods of progression.. Eur Spine J.

[10] Haumont T, Gauchard GC, Lascombes P, Perrin PP (2011). Postural instability in early-stage idiopathic scoliosis in adolescent girls.. Spine (Phila Pa 1976).

[11] Stylianides GA, Beaulieu M, Dalleau G, Rivard CH, Allard P (2011). Iliac Crest Orientation and Geometry in Able-bodied and Non-treated Adolescent Idiopathic Scoliosis Girls with Moderate and Severe Spinal Deformity.. European Spine Journal In press.

[12] Nicolopoulos KS, Burwell RG, Webb JK (1985). Stature and its components in adolescent idiopathic scoliosis. Cephalo-caudal disproportion in the trunk of girls.. J Bone Joint Surg Br.

[13] Pasha S, Sangole AP, Aubin CE, Parent S, Mac-Thiong JM (2010). Characterizing pelvis dynamics in adolescent with idiopathic scoliosis.. Spine (Phila Pa 1976).

[14] Goldberg CJ, Kaliszer M, Moore DP, Fogarty EE, Dowling FE (2001). Surface topography, Cobb angles, and cosmetic change in scoliosis.. Spine (Phila Pa 1976).

[15] Thulbourne T, Gillespie R (1976). The rib hump in idiopathic scoliosis. Measurement, analysis and response to treatment.. J Bone Joint Surg Br.

[16] Ramirez L, Durdle NG, Raso VJ, Hill DL (2006). A support vector machines classifier to assess the severity of idiopathic scoliosis from surface topography.. IEEE Trans Inf Technol Biomed.

[17] Burwell RG, Dangerfield PH, Vernon CL (1981). Bone asymetry and joint laxity in the upper limbs of children with adolescent idiopathic scoliosis.. Ann R Coll Surg Engl.

[18] Gum JL, Asher MA, Burton DC, Lai SM, Lambart LM (2007). Transverse plane pelvis rotation in adolescent idiopathic scoliosis: primary or compensatory?. Eur Spine J.

[19] Sahlstrand T, Petruson B, Ortengren R (1979). Vestibulospinal reflex activity in patients with adolescent idiopathic scoliosis. Postural effects during caloric labyrinthine stimulation recorded by stabilometry.. Acta Orthop Scand.

[20] Bunnell WP (1986). The natural history of idiopathic scoliosis before skeletal maturity.. Spine (Phila Pa 1976).

[21] Isu T, Chono Y, Iwasaki Y, Koyanagi I, Akino M (1992). Scoliosis associated with syringomyelia presenting in children.. Childs Nerv Syst.

[22] Lonstein JE, Carlson JM (1984). The prediction of curve progression in untreated idiopathic scoliosis during growth.. J Bone Joint Surg Am.

[23] Morrissy RT, Goldsmith GS, Hall EC, Kehl D, Cowie GH (1990). Measurement of the Cobb angle on radiographs of patients who have scoliosis. Evaluation of intrinsic error.. J Bone Joint Surg Am.

[24] Wong HK, Tan KJ (2010). The natural history of adolescent idiopathic scoliosis.. Indian J Orthop.

[25] McIlroy WE, Maki BE (1997). Preferred placement of the feet during quiet stance: development of a standardized foot placement for balance testing.. Clin Biomech (Bristol, Avon).

[26] LeBlanc R, Labelle H, Rivard CH, Poitras B (1997). Relation between adolescent idiopathic scoliosis and morphologic somatotypes.. Spine (Phila Pa 1976).

[27] Bellefleur C, Labelle H, Dansereau J, de Guise J, Stokes IA (1994). [Intraoperative three-dimensional evaluation of Cotrel-Dubousset’s procedure for the treatment of idiopathic scoliosis].. Ann Chir.

[28] Dalleau G, Damavandi M, Leroyer P, Verkindt C, Rivard CH (2011). Horizontal body and trunk center of mass offset and standing balance in scoliotic girls.. Eur Spine J.

[29] Simoneau M, Mercier P, Blouin J, Allard P, Teasdale N (2006). Altered sensory-weighting mechanisms is observed in adolescents with idiopathic scoliosis.. BMC Neurosci.

[30] Maki BE, Holliday PJ, Topper AK (1994). A prospective study of postural balance and risk of falling in an ambulatory and independent elderly population.. J Gerontol.

[31] Dalleau G, Allard MS, Beaulieu M, Rivard CH, Allard P (2007). Free moment contribution to quiet standing in able-bodied and scoliotic girls.. Eur Spine J.

[32] Holland BS, Copenhaver M (1988). Improved Bonferroni-type multiple testing procedures.. Psychol Bull.

[33] Archer IA, Dickson RA (1985). Stature and idiopathic scoliosis. A prospective study.. J Bone Joint Surg Br.

[34] Lowe TG, Edgar M, Margulies JY, Miller NH, Raso VJ (2000). Etiology of idiopathic scoliosis: current trends in research.. J Bone Joint Surg Am.

[35] Samuelsson L, Noren L (1997). Trunk rotation in scoliosis. The influence of curve type and direction in 150 children.. Acta Orthop Scand.

[36] Amendt LE, Ause-Ellias KL, Eybers JL, Wadsworth CT, Nielsen DH (1990). Validity and reliability testing of the Scoliometer.. Phys Ther.

[37] Mubarak SL, Wyatt MP, Leach J (1985). Evaluation of the intraexaminer and interexaminer reliability of the scoliometer in measuring trunk rotation.. Orthop Trans.

[38] Dickson RA, Sevitt EA (1982). Growth and idiopathic scoliosis: a longitudinal cohort study.. J Bone Joint Surg Br.

